# Compression fossil Mymaridae (Hymenoptera) from Kishenehn oil shales, with description of two new genera and review of Tertiary amber genera

**DOI:** 10.3897/zookeys.130.1717

**Published:** 2011-09-24

**Authors:** John T. Huber, Dale Greenwalt

**Affiliations:** 1 Natural Resources Canada, c/o AAFC, K.W. Neatby Building, 960 Carling Ave., Ottawa, ON, K1A 0C6, Canada; 2 Paleobiology Department, National Museum of Natural History, Washington, DC, 20013, USA

**Keywords:** Mymaridae, Kishenehn Formation, compression fossils, Eoanaphes, Eoeustochus, Gonatocerus, Baltic amber

## Abstract

Compression fossils of three genera and six species of Mymaridae (Hymenoptera: Chalcidoidea) are described from 46 million year old Kishenehn oil shales in Montana, USA. Two new genera are described: *Eoeustochus* Huber, **gen. n.**, with two included species, *Eoeustochus kishenehn* Huber (type species) and *Eoeustochus borchersi* Huber, **sp. n.**, and *Eoanaphes*, **gen. n.**, with *Eoanaphes stethynioides* Huber, **sp. n.** Three new species of *Gonatocerus* are also described, *Gonatocerus greenwalti* Huber, **sp. n.** , *Gonatocerus kootenai* Huber, **sp. n.**, and *Gonatocerus rasnitsyni* Huber, **sp. n.** Previously described amber fossil genera are discussed and five genera in Baltic amber are tentatively recorded as fossils: *Anagroidea*, *Camptoptera*, *Dorya*, *Eustochus*, and *Mimalaptus*.

## Introduction

The family Mymaridae (Hymenoptera: Chalcidoidea) is represented by 103 genera and about 1400 nominal extant species in all terrestrial habitats and a few fresh water habitats (Huber 1986, Noyes 2010). Fossil Mymaridae have been described from amber inclusions from various localities, including five extinct genera and species from Cretaceous amber (Yoshimoto 1975, Huber and Poinar 2011) and eight genera (seven still extant, one extinct) from Tertiary amber, mostly from the Samland Peninsula, Kaliningrad district, Russia, and Chiapas, Mexico. Probably because of their small size, no compression fossils of Mymaridae have been discovered until now. Here we describe the first ones, representing three genera and six species. These and the Tertiary amber fossil genera are discussed and compared with extant genera.

## Methods

A total of 17 specimens of fossil Mymaridae, all females, were collected in 2009 and 2010 at six sites (e.g. 113°42.173'W, 48°23.476'N) along the Middle Fork of the Flathead River in northwestern Montana between Paola and Coal Creeks in accordance with USFS Authorization HUN281. Fossil specimens were collected from the middle sequence of the Coal Creek member of the Kishenehn Formation, which has been estimated to be 46.2 +/- 0.4 myo (Lutetian) by ^40^Ar/^39^Ar analysis and 43.5 +/- 4.9 myo by fission-track analysis (Constenius 1996). Although the paper/oil shales of the middle sequence are thin (<1 mm to several mm), they were often easily split into even thinner pieces so as to expose unweathered surfaces on which the fossil insects reside.

The compression fossils were immersed in 95% ethanol for examination and photography. For figures 1–10 and 13–18, specimens were photographed using a Zeiss AxioCam MRc5 digital CCD camera mounted on a Zeiss Discovery V20 microscope and Zeiss AxioVision EDF software in Ottawa, ON. Measurements were generated using Zeiss AxioVision software. For figures 11 and 12, specimens were photographed using an Olympus SZX12 microscope, DP-25 camera and DPM imaging software in Washington, DC, and measurements were taken with the DP2-BSW software. All measurements are in micrometers (mm). If a measurement could not be made (e.g., scape length) it is represented by a dash.

Abbreviations used: fl_x _= funicle segment x. Measurements were taken as accurately as possible but given that the beginning and end points of a structure were not always clear or were hidden, the measurements may not be accurate. Appendage measurements are the most accurate except the wing bases cannot be clearly determined. Consequently, wing lengths were taken from the visible edge of the mesosoma and therefore they and length/width ratios are slightly smaller than they should be.

Fossils examined are in the following institutions:

AMNH American Museum of Natural History.

IPMGö Institute and Museum für Paläontologie, Georg-August University, Göttingen, Germany.

NMNH National Museum of Natural History, Washington, D.C.

OSU Oregon State University, G. Poinar, Jr. collection, Corvallis, Oregon, USA.

SVT S. V. Triapitsyn private collection, California, USA.

UCRC University of California, Riverside, California, USA.

ZMUC Zoological Museum, University of Copenhagen, Denmark.

The compression fossils are housed in NMNH. Baltic amber fossils were examined from the remaining institutions.

## Systematics

### Eoeustochus

Huber gen. n.

urn:lsid:zoobank.org:act:25AE7CE1-EB1D-4B39-8D61-AA86366A4A35

http://species-id.net/wiki/Eoeustochus

**Figures 1–2.**
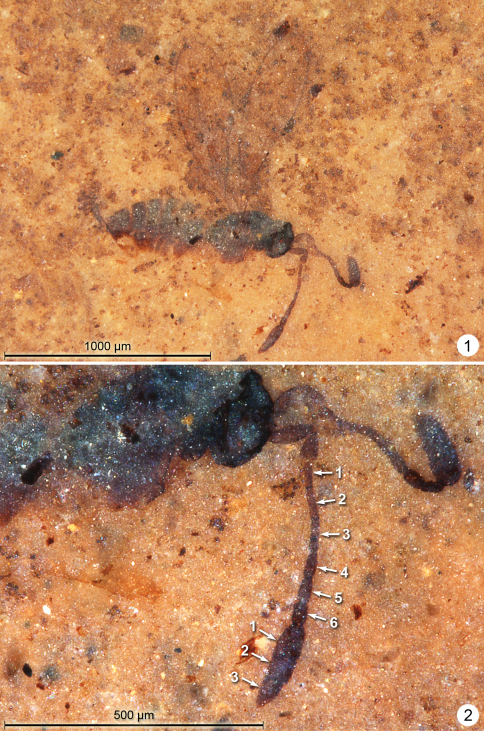
*Eoeustochus kishenehn*, holotype **1** habitus lateral **2** mesosoma, head, antennae.

Figs 1 [Fig F2] [Fig F3] 8

#### Description.

**Female.** Body length 718–1133 (seven specimens in total, only four described and named to species). **Head.** Normal in shape, wider than long and about ¾ as high as wide. Face slightly convex in lateral view; vertex flat and slightly sloping anteriorly, forming a moderately sharp angle with occiput; back of head slightly concave. Eye higher than wide, about 2/3 head height; malar space about 1/3 eye height. **Antenna.** Funicle 6-segmented, with each funicle segment longer than wide; clava 3-segmented with the claval sutures perpendicular to claval length. **Wings.** Fore wing wide, symmetrical, with evenly rounded apex (shape resembling that of the extant genus *Eustochus*); marginal fringe shorter than fore wing width. Venation about 0.4× fore wing length, with long marginal vein and short but distinct stigmal vein. Hind wing narrow; marginal fringe much longer than wing width. **Mesosoma.** Shorter than gaster. Pronotum length at most about half length of mesoscutum. Mesoscutum length subequal to scutellum. Scutellum with frenum apparently entire, not divided longitudinally. Metanotum much shorter than scutellum. **Metasoma.** Constricted at base, probably with short petiole. Gastral terga similar in length. Ovipositor moderately short, probably arising near midpoint of gaster and its apex not or barely exserted beyond gastral apex.

#### Type species.

*Eoeustochus kishenehn* Huber, **sp. n.**

#### Derivation of generic name.

The name is from eo-, Greek for early, and *Eustochus*, an extant genus. The gender is masculine.

#### Discussion. 

Two species are described, each from two specimens. Three additional specimens (Kishenehn #30,356, 40,410, and 40,023) belong to *Eoeustochus* but are not included in the type series of either species because they are not in as good a condition.

The relationships of *Eoeustochus* are with *Eustochus*, one extant species of which has a 3-segmented clava in females. Although thirteen extant genera have a definite 3-segmented clava (*Allanagrus*, *Anneckia*, *Idiocentrus*, *Krokella*, *Nesopatasson/Nesomymar*, *Neostethynium*, *Notomymar*, *Paracmotemnus*, *Parastethynium*, *Polynemoidea*, *Pseudocleruchus*, *Stethynium*), the closest one is *Eustochus*, based on the apparent strong constriction in lateral view between meso- and metasoma (none in *Allanagrus*), transverse sutures between claval segments (sutures oblique in *Stethynium*), shape of fully developed wings (wings almost absent in *Nesopatasson*/*Nesomymar* and *Notomymar*, and of different shape in *Anneckia*, *Neostethynium*, *Parastethynium* and *Pseudocleruchus*) venation shorter than half fore wing length (venation longer in *Krokella* and *Paracmotemnus*), and ovipositor not distinctly exserted beyond apex of gaster (ovipositor strongly exserted in *Polynemoidea*) or projecting anteriorly under mesosoma (ovipositor strongly projecting anteriorly in *Idiocentrus*).

### Eoeustochus kishenehn

Huber sp. n.

urn:lsid:zoobank.org:act:5CE2A3C6-9203-4A2A-8B2F-4FECC0937342

http://species-id.net/wiki/Eoeustochus_kishenehn

Figs 1 4

#### Type material.

Holotype female (NMNH), labelled “*Eoeustochus kishenehn* Huber Holotype female #543757”.

Paratype female (NMNH), labelled as for holotype but “paratype #543758”.

#### Description.

**Figures 3–4. F2:**
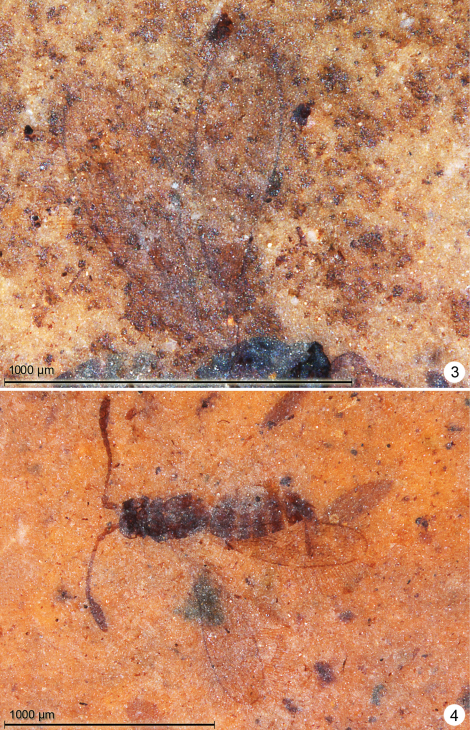
*Eoeustochus kishenehn* **3** wings, holotype **4** paratype, habitus dorsal.

**Female.** Colour brown, head dark brown, fore wing thinly margined with brown. Holotype (Fig. 1) measurements as follows. Body length 909. Antenna (Fig. 2) with total funicle length 317; scape 95, pedicel 70, fl_1 _52, fl_2_ 63, fl_3_ 63, fl_4_ 51, fl_5_ 47, fl_6_ 45; clava 180, 0.56× funicle length. Fl_2_ and fl_3 _the longest segments and fl_6_ the shortest (Fig. 2). Mesosoma length 481. Fore wing (Fig. 3) length 912, width 271, length/width 3.36, longest marginal setae 160, venation length 355. Hind wing width 26, longest marginal setae 88. Metasoma length 340. Ovipositor length 290, apparently slightly exserted beyond apex of gaster.

Paratype (Fig. 4) measurements as follows. Body length 966, head width 234, height 132.

#### Derivation of species name.

Named after the Kishenehn Formation shale in which the fossils were found.

### Eoeustochus borchersi

Huber sp. n.

urn:lsid:zoobank.org:act:B96FABBE-964F-4D92-AF7A-D3DE8EBB780B

http://species-id.net/wiki/Eoeustochus_borchersi

Figs 5 8

#### Type material.

Holotype female (NMNH ) labelled “*Eoeustochus borchersi* Huber Holotype female #543759”

Paratype female (NMNH), labelled as for holotype but “paratype #543760”.

#### Description.

**Figures 5–6. F3:**
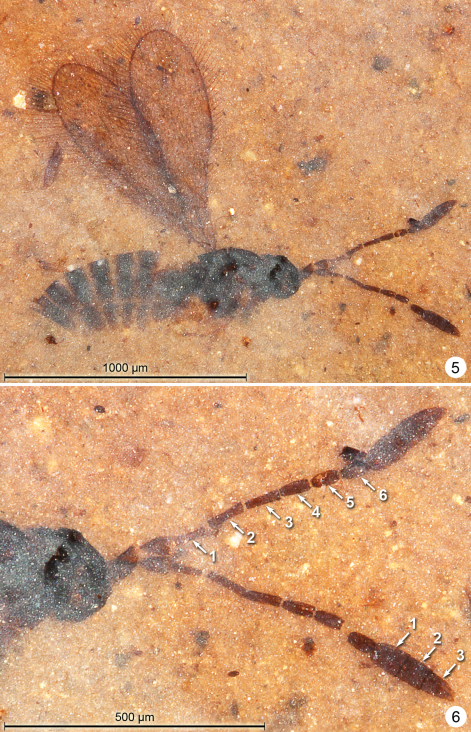
*Eoeustochus borchersi*, holotype **5** habitus lateral **6** head and antennae.

**Figures 7–8.**
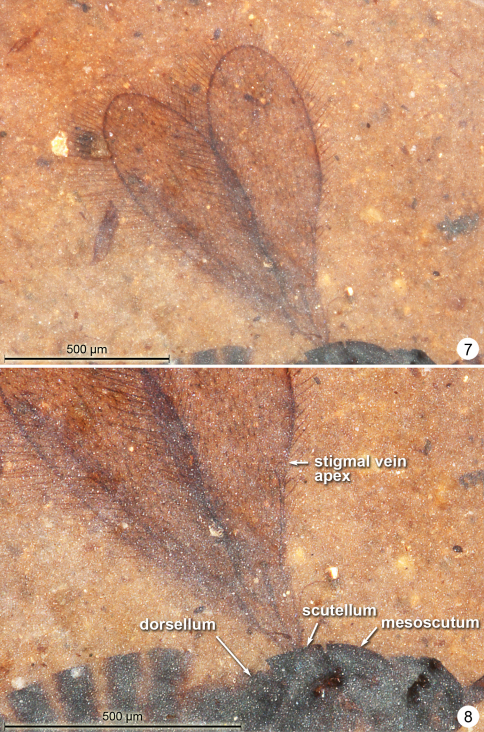
*Eoeustochus borchersi*, holotype **7** wings **8** mesosoma and base of wings.

**Female.** Colour dark brown, fore wing thinly margined with brown. Holotype (Fig. 5) measurements as follows. Body length 1094. Antenna (Fig. 6) with total funicle length 432; scape -, pedicel 58, fl_1_ 69, fl_2_ 78, fl_3_ 71, fl_4_ 64, fl_5_ 56, fl_6_ 54; clava 186, 0.47× funicle length. Fl_2_ distinctly the longest segment and fl_6_ the shortest. Fore wing (Fig. 7) length 948, width 312, length/width 2.72, longest marginal setae 188, venation (Fig. 8) length 395. Hind wing length 781, width 32. Ovipositor length 292, not exserted beyond apex of gaster.

#### Comments.

This species differs from *Eoeustochus kishenehn* by the longer funicle segments (funicle 2.31× clava length cf. 1.83× in *Eoeustochus kishenehn*) and slightly wider fore wing (length/width at most 3.49 instead of at least 3.64 in *Eoeustochus kishenehn*).

#### Derivation of species name.

Named after Harold Borchers, professor of entomology and early mentor to the junior author at Bemidji State University, Bemidji, Minnesota.

### Gonatocerus

Nees

**Figures 9–10.**
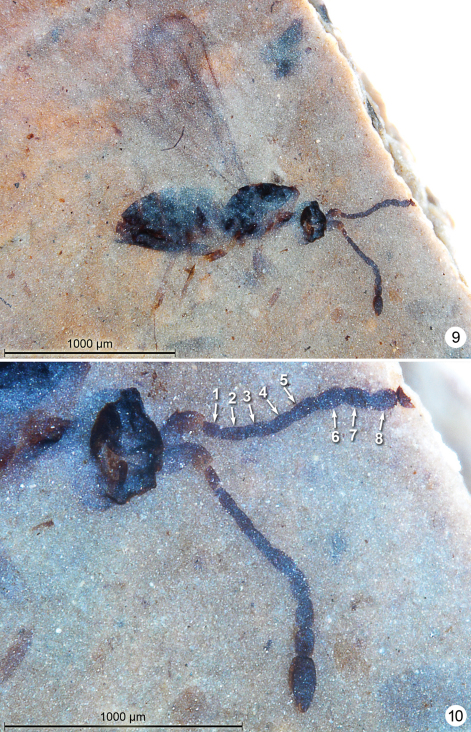
*Gonatocerus kootenai*, holotype **9** habitus lateral **10** head and antennae.

Figs 9 [Fig F6] [Fig F7] 16

*Gonatocerus* is a worldwide group with numerous described extant species classified in several subgenera (Triapitsyn et al. 2010). Its members are often the most commonly collected Mymaridae in almost any habitat so it seems surprising that it has been recorded only once as a fossil, in Baltic amber by Meunier (1905).

The three species described below definitely belong to *Gonatocerus* based onthe entire clava, 8-segmented funicle, similar sized gastral segments, short and probably narrow petiole, and 5-segmented tarsi (at least in the one species where they can be counted). Because they all appear to have a rhomboidal dorsellum they would be classified either in *Gonatocerus (Gonatocerus)* or in *Gonatocerus (Cosmocomoidea)*, but not *Gonatocerus (Lymaenon)*, the most common extant subgenus because it has a narrow, strap-shaped dorsellum or *Gonatocerus (Gastrogonatocerus)* because the ovipositor does not project forward under the mesosoma.

### Gonatocerus kootenai

Huber sp. n.

urn:lsid:zoobank.org:act:5FF0E145-6ABD-431A-A540-8FEC3130E934

http://species-id.net/wiki/Gonatocerus_kootenai

Figs 9 11

#### Type material.

Holotype female (NMNH) labelled “*Gonatocerus kootenai*Huber Holotype female #543761”.

#### Description.

**Figures 11–12. F6:**
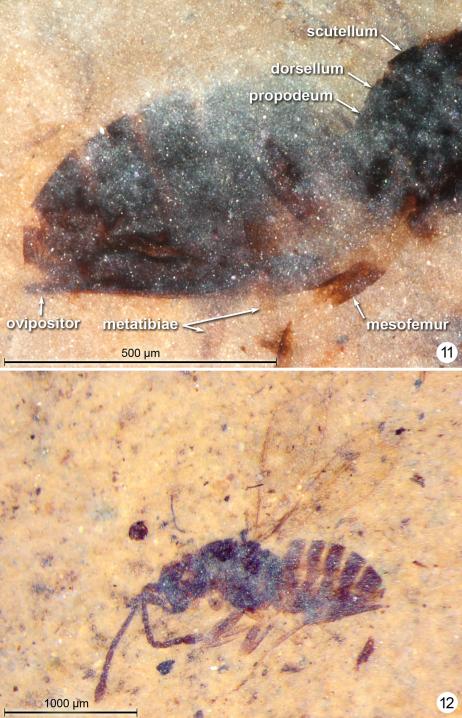
*Gonatocerus* spp. **11** *Gonatocerus kootenai* holotype, posterior half of mesosoma and metasoma **12** *Gonatocerus rasnitsyni* holotype, habitus lateral.

**Female.** Colour dark brown except pedicel laterally, pronotum, trochanters, base and apex of femora, base and apex of tibiae (at least of fore and middle legs) and tarsomeres except apical tarsomere of all legs whitish. Holotype (Fig. 9) measurements as follows. Body length 1270. Head height 246, length 157. Antenna (Fig. 10) with total funicle length 476; scape -, pedicel 57, fl_1_ 54, fl_2_ 57, fl_3_ 52, fl_4_ 59, fl_5_ 69, fl_6_ 64, fl_7_ 61, fl_8_ 60, clava 120. Mesosoma length 462. Fore wing length 1101, width 309, length/width 3.56, venation length 259, longest marginal setae 95. Hind wing length 729, width 35, longest marginal setae 67. Metasoma (Fig. 11) length 658. Ovipositor length 588.

#### Comments.

*Gonatocerus kootenai* differs from the following two species by the relatively narrower wings and shorter funicle segments. I tentatively place it in *Gonatocerus (Gonatocerus)* because of the relatively narrow fore wing and fairly long and relatively slender fl_3_–fl_5. _The reverse side of the shale piece contains a specimen of Trichoptera.

#### Derivation of species name.

Named after the Kootenai tribe of the Flathead Nation in northwestern Montana, site of the Kishenehn shales.

### Gonatocerus rasnitsyni

Huber sp. n.

urn:lsid:zoobank.org:act:7F2CF9FB-5B06-4ABF-B62B-6043AB4179C7

http://species-id.net/wiki/Gonatocerus_rasnitsyni

Figs 12 13

#### Type material.

Holotype female (NMNH) labelled “*Gonatocerus rasnitsyni* Huber Holotype female #543762”.

#### Description.

**Figures 13–14. F7:**
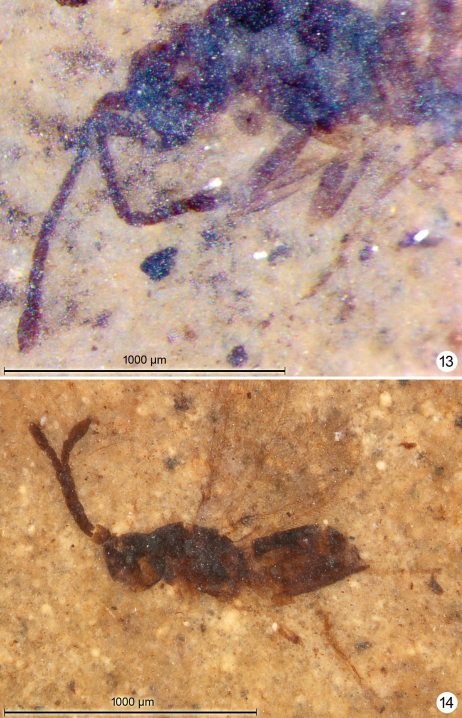
*Gonatocerus* spp. **13** *Gonatocerus rasnitsyni* holotype, mesosoma, head, antennae **14** *Gonatocerus greenwalti* holotype, habitus lateral.

**Female.** Colour dark brown except middle leg (others less clearly visible) with coxa, trochanter, apex of femur and base of tibia, and basal 4 tarsal segments yellowish. Holotype (Fig. 12) measurements as follows (measured in NMNH only).Body length 1046. Antenna (Fig. 13) with total funicle length 414: scape length and width 144/46, pedicel -, fl_1_ 44, fl_2_ 43, fl_3_ 55, fl_4_ 51, fl_5_ 65, fl_6_ 56, fl_7_ 58, fl_8_ 50, clava 113. At least on one antenna it appears that fl_3_ is wider than fl_2_ and fl_4. _Mesosoma length (excluding pronotum) 375. Fore wing length 789 (775 from margin of mesosoma), width 243, length/width 3.25.

#### Comments.

*Gonatocerus rasnitsyni* differs from *Gonatocerus kootenai* by the relatively smaller size, shorter and wider fore wing, and colour of the antennal pedicel and legs. I tentatively place it in *Gonatocerus (Cosmocomoidea)* because of the relatively wider fl_3 _(on one antenna at least, suggesting it bears multiporous plate sensilla) compared to the slightly narrower fl_2_ and fl_4. _This resembles several extant members of this subgenus that also have alternately wider and narrower basal funicle segments.

#### Derivation of species name.

Named in honour of A. P. Rasnitsyn, the world’s foremost Hymenoptera palaeontologist, on the occasion of his 75^th^ birthday.

### Gonatocerus greenwalti

Huber sp. n.

urn:lsid:zoobank.org:act:957095F5-B63B-4FB5-9FF0-0ACD1B029730

http://species-id.net/wiki/Gonatocerus_greenwalti

Figs 14 16

#### Type material.

Holotype female (NMNH) labelled “*Gonatocerus greenwalti* Huber Holotype female #543763”.

#### Description.

**Figures 15–16.**
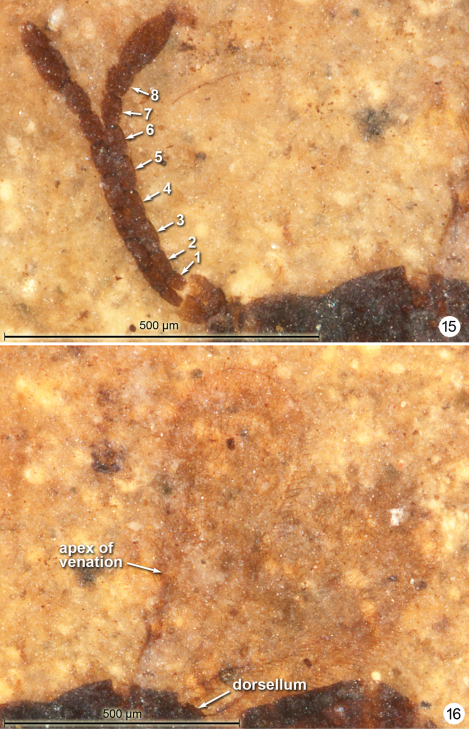
*Gonatocerus greenwalti* holotype: antennae **16** wings.

**Female.** Colour dark brown except apex of pedicel and legs beyond coxae lighter (yellowish). Holotype (Fig. 14) measurements as follows.Body length 926. Antenna (Fig. 15) with total funicle length 398; scape -, pedicel 51, fl_1_ 35, fl_2_ 42, fl_3_ 57, fl_4_ 54, fl_5_ 56, fl_6_ 55, fl_7_ 56, fl_8_ 54, clava 116. Mesosoma length 410. Fore wing (Fig. 16) length 730, width 273, length/width 2.67, longest marginal setae 58. Fore wing seemingly bare (without microtrichia) behind and just apical to the venation. Hind wing length 458, width 23, longest marginal setae 73. Metasoma length 460. Ovipositor length 417.

#### Comments.

*Gonatocerus greenwalti* differs from *Gonatocerus kootenai* and *Gonatocerus rasnitsyni* by the wider fore wing and thicker funicle segments. The apparent absence of microtrichia behind the venation, the wide fore wing, and fairly uniformly thick funicle segments suggest that *Gonatocerus greenwalti* should be classified in *Gonatocerus (Cosmocomoidea)*. The shale fragment in which the fossil occurs contains several aquatic insects (e.g., Notonectidae), an indication of the lacustrine environment in which the mymarid lived (though it is not aquatic itself).

#### Derivation of species name.

Named by the senior author in honour of the junior author, Dale Greenwalt, who collected and curated the insect fossils from Kishenehn shale.

### Eoanaphes

Huber gen. n.

urn:lsid:zoobank.org:act:1DF7117D-B881-487B-94D8-63BCAA207F8F

http://species-id.net/wiki/Eoanaphes

Figs 17, 18

#### Description.

**Female.** Body about 700. **Head.** About as wide as high (measured in frontal view). Malar space long, almost eye height. **Antenna.** Funicle 6-segmented, with fl_1_ very short and remaining segments longer than wide; clava 3-segmented with the claval sutures almost perpendicular to claval length. **Wings.** Fore wing wide, slightly asymmetrical, with somewhat truncate apex; marginal fringe much shorter than fore wing width. Venation almost 1/3 fore wing length, with marginal vein fairly short, about as long as stigmal vein. Hind wing narrow; marginal fringe much longer than wing width and along posterior margin extending to base of membrane.

#### Mesosoma.

About 0.75 x gaster length. Mesoscutum length shorter than scutellum. Metanotum much shorter than scutellum, apparently with strap-like dorsellum. **Metasoma.** Apparently slightly constricted at base, probably with fairly wide, short petiole. Gastral terga similar in length. Ovipositor moderately short, apparently arising near base of gaster and its apex not exserted beyond gastral apex.

**Figures 17–18.**
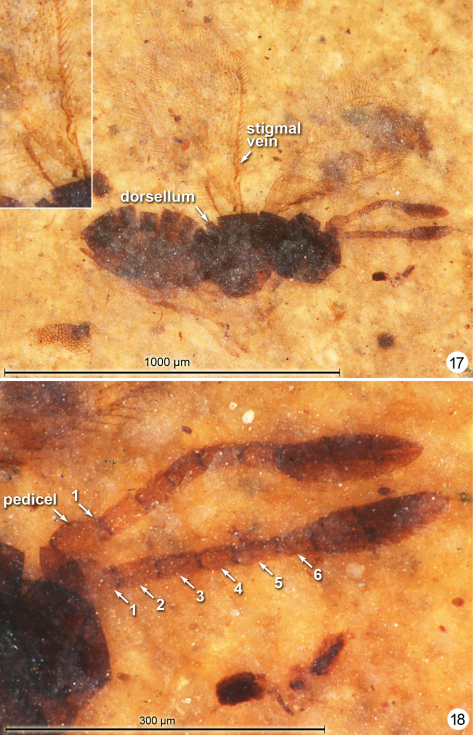
*Eoanaphes stethynioides*, holotype **17** habitus lateral (insert fore wing base) **18** antennae.

#### Type species.

*Eoanaphes stethynioides* Huber, sp. n.

#### Derivation of generic name.

The name is based on the extant genus *Anaphes*, which is also known from one extinct species in Baltic amber. The gender is masculine.

#### Discussion.

*Eoanaphes* appears to be related to two genera, *Anaphes* and *Stethynium*. Four features suggest *Anaphes*: 1) in lateral view there is a distinct constriction dorsally between the mesosoma and metasoma, indicating that the propodeum slopes strongly down relative to the horizontal scutellum and dorsellum (in *Stethynium*, the entire dorsal margin of the mesosoma is horizontal and in line with the base of the metasoma, without a depression between the two parts); 2) the very short fl_1_; 3) the apparent absence of a distinct rounded lobe on the posterior margin of the fore wing opposite the marginal + stigmal veins (one wing appears to have a rounded lobe but this is due to the membrane being partly folded over on itself); 4) the wing surface behind the marginal vein seems to be bare except for a few setae behind the stigmal vein and a faint suggestion of a hair line separating medial from marginal space. Two features suggest *Stethynium*: 1)the clearly 3-segmened clava; 2) the strap-like dorsellum (rhomboidal in *Anaphes*).

### Eoanaphes stethynioides

Huber sp. n.

urn:lsid:zoobank.org:act:653B7B5B-8401-42A6-9790-826E714E3C22

http://species-id.net/wiki/Eoanaphes_stethynioides

Figs 17, 18

#### Type material.

Holotype female (NMNH) labelled “*Eoanaphes stethynioides* Huber Holotype female #543764”.

#### Description.

**Female.** Colour dark brown; pedicel, basal two funicle segments, and tarsi except perhaps apical tarsomere yellowish. Holotype (Fig. 17) measurements as follows.Bodylength 703. Head width 199. Antenna (Fig. 18) with total funicle length 189; scape -, pedicel 50, fl_1_ 15, fl_2_ 38, fl_3_ 38, fl_4_ 34, fl_5_ 40, fl_6_ 34, clava 132, about 0.66× funicle length. Mesosoma length (excluding pronotum) 293. Fore wing length 593, width 248, length/width 2.39, longest marginal setae 99; venation length 169, about 0.28× fore wing length. Hind wing length 485, width 34, venation length 115, longest marginal setae 84. Gaster length 372. Ovipositor length 322.

## Discussion

Compression fossils in sedimentary rock are less informative morphologically than amber fossils. Fine details such as body setation and sculpture are not visible (wing setae may be visible), and body flattening during fossilization usually results in distortion. Measurements of body parts may therefore not be accurate though they can sometimes be more easily measured because they are flat. Appendages are the best source of useful diagnostic characters because some (legs, antenna) are less and some (wings) are not distorted. Because of the preservation method, compression fossils cannot be easily compared to amber fossils or extant genera and species. Nonetheless, they can be diagnosed moderately well and differentiated with reasonable certainty from each other and from other extinct and extant genera.

Among the 17 Kishenehn mymarids found, *Eoeustochus* is the most common, followed by *Gonatocerus* and *Eoanaphes*. It is surprising that no males were found; in extant genera males are less common than females but they are rarely almost completely absent. The compression fossils are of middle Eocene (Lutetian) age, as indicated above, and so are most Baltic amber fossils (Weitschat and Wichard 2010), i.e., about 41–49 my old. Incidentally, Meunier (1901), quoting A. Jentzsch, had noted that their age was lower Eocene, not lower Oligocene (yet the latter was stated again by Meunier 1909 and Doutt 1973). The Eocene had a warm humid climate, significantly warmer than the present (Zachos et al. 2001), allowing for development of subtropical and tropical rain forests to which the Baltic amber forests belong (Weitschat and Wichard 2010). Baltic amber forests were apparently rich in lentic water, flood plains, ponds, lakes with littoral habitats and temporary micro waters. Aquatic insects make up 25% of Baltic amber inclusions (Weitschat and Wichard 2010). The Middle sequence of the Coal Creek member of the Kishenehn Formation consisted of lacustrine sediments of a large permanent and shallow lake and associated lakeside and/or marsh, and has a high proportion of aquatic insect fossils (e.g., > 60% of all the fossil insects are Corixidae and Chironomidae). Because the climate and habitat of both Baltic and Kishenehn fossils was apparently similar it is not surprising that the genera of Mymaridae found in each are similar.

Meunier (1901) described most Baltic amber fossils of Mymaridae. Doutt (1973) described a few species from Mexican amber (Simojovel, Chiapas), dated as 15–20 my, and Thuróczy (1983) described one species. There is no indication from morphology that any of these or the mymarid compression fossils form a link between Cretaceous and Tertiary/Quaternary genera and species. Instead, they add support to the analysis by Rasnitsyn and Kulicka (1990) that showed Hymenoptera assemblages from Baltic amber seem to be more similar to the extant fauna than to the Late Cretaceous fauna.

One clear morphological tendency from Cretaceous to Tertiary and Quaternary (present) species can be seen — a reduction in the number of funicle or claval segments in females. A 3-segmented clava occurs in three of five (60%) Cretaceous genera, four of ten (40%) previously reported Tertiary genera from amber and shale (reported above), and 13 of 103 (about 8%) currently recognized extant genera. An 8-segmented funicle occurs in three of five (60%) Cretaceous genera, one of the 10 (10%) previously reported Tertiary genera, and five of the 103 (about 5%) extant genera. Except for *Gonatocerus*, which has eight funicle segments but an entire clava, all other Tertiary fossils have six (rarely fewer) funicle segments in females.

Spahr (1987) catalogued the literature and listed 11 mymarid genera from amber, three of them Cretaceous and eight Tertiary. He followed previous authors by including genera and species now correctly classified in Mymarommatidae (Gibson et al. 2007). Witsack (1986) described Palaeopatasson for one species from Dominican amber.

The senior author examined 16 Baltic amber pieces (SVT, UCRC) in 2005, 13 pieces (AMNH) in 2011, and three pieces (IPMGö., ZMUC) in about 2005. Only a few specimens could be referred confidently to an extant genus. These were four specimens of *Gonatocerus*, and (less confidently) three specimens of *Anaphes* and two of *Stethynium*. If correctly identified, five more genera, all known from the extant fauna, are reported here for the first time as amber fossils. Having examined these 32 additional amber specimens and realizing that most of them cannot definitely be classified in an extant genus, the senior author has doubts about the correct generic placement of at least some of the specimens studied by past workers. None of those specimens were examined, however. Each Tertiary fossil genus is discussed briefly below.

*Anagroidea* (1 female, C.V. Henningsen, B-1 1956, ZMUC, examined) is no longer known from extant species in Europe and is rare in the Holarctic region (eastern Asia and south east USA).

*Alaptus* is known from at least two species in Miocene amber (Doutt 1973) that are probably correctly identified to genus.

*Anaphes* was known from three specimens in Baltic amber. *Anaphes splendens*Meunier is probably incorrectly classified. According to Meunier the ovipositor extends appreciably beyond the apex of the metasoma, unlike any extant *Anaphes*. The fore wing with a slight but distinct ventral lobe, its overall shape, the venation with a distinct stigmal vein, and the antenna with fl_1_ almost as long as fl_2_ also do not fit the genus. *Anaphes schellwieniens* Meunier is described from a male so it is impossible to determine its placement. Doutt (1973) examined a male from Chiapas amber and tentatively placed it in *Anaphes*. Three specimens examined here are fairly confidently placed in *Anaphes* (1 female, UCRC; 2 females, SVT). Additional specimens (2 females, SVT) examined are tentatively placed in *Anaphes*.

*Arescon* is known from two species in amber (Meunier 1901, 1905) that are probably correctly placed because of their 5-segmented funicle, though fore wing venation length, which is important information for correct generic placement, was not given by Meunier.

?*Camptoptera* (1 female, SVT), examined in 2005, if correctly classified, would be the first fossil specimen for this worldwide genus.

?*Dorya* (1 female, UCRC) is known only from extant species in Australia and New Zealand.

?*Eustochus* (1 female, AMNH). The gaster appears to be petiolate. This suggests a *Eustochus* with a distinct 3-segmented clava (most have a 2-segmented clava) though the wing shape and antenna suggests *Eoeustochus*.

?*Mimalaptus* (3 females, SVT; 2 females, UCRC; 1 female, box G 3.910 Hymenoptera #BST03124, IPMGö.) is known from extant species in Australia and New Zealand and possibly eastern Asia.

*Gonatocerus* was known from only one fossil specimen, *Gonatocerus henneberti* (Meunier 1905), which appears to belong to either *Gonatocerus (Gonatocerus)* or *Gonatocerus (Lymaenon)* as suggested by the narrow wings. However, the “écusson semilunaire” [semilunate shield], which I interpret from Meunier’s drawing as being the dorsellum, appears to be rhomboidal in shape, which would eliminate *Gonatocerus (Lymaenon)* as the correct subgenus. The small size of the specimen, only 1/3 mm long, is unusual; it is much smaller than the smallest extant *Gonatocerus* I have seen. A specimen of *Gonatocerus (Cosmocomoidea)* is reported here (1 male, Poinar collection, OSU) from Dominican amber, 15–20 my.

*Litus* is represented by two fossil species. *Litus elegans* Meunier (1901) may or may not be correctly classified because the description and illustration are insufficient to place it. It apparently differs considerably from *Litus mexicanus* Doutt (1973), which is likely correctly classified. Extant *Litus* species tend to have a bi-geniculate funicle, unlike that illustrated by Meunier. *Litus beneficus* Meunier from copal (recent) from Madagascar also needs to be re-examined; it is unlikely to be classified correctly.

*Malfatia molitorae* Meunier (1901) is based on a male and from the description alone it is impossible to determine how it relates to extant genera. Its status remains uncertain.

*Palaeopatasson grollei* Witsack (1986) is based on a female and may be related to Anaphes.

*Polynemoidea mexicana* Doutt (1973) from Chiapas amber was stated to be similar to *Polynemoidea domestica* Girault, but the latter species is likely incorrectly placed in *Polynemoidea* (Lin et al. 2010). Consequently, I suspect that *Polynemoidea mexicana* is also incorrectly classified; it does not have a strongly exserted ovipositor as in the type species of *Polynemoidea*.

*Stethynium* (1 female, S. Triapitsyn collection), if correctly identified, confirms that the genus occurs in Baltic amber, as reported by Thuróczy (1983).

## Conclusions

1. Tertiary fossils of Mymaridae are clearly more related to the extant fauna than to the Cretaceous fauna.

2. Except for *Gonatocerus* and the two new genera described above, tertiary fossils of Mymaridae (except perhaps *Anaphes* and *Stethynium*) are for the most part doubtfully assigned to extant genera.

3. Several Eocene amber fossils, if correctly identified to genus, represent genera that no longer occur in Europe (*Anagroidea*, *Dorya*, *Mimalaptus*) or are very poorly represented in most of the Holarctic region (*Stethynium*). These genera are now distributed mostly in the tropics or southern hemisphere, supporting observations that the Eocene climate (in parts of the Holarctic region at least) was considerably warmer than at present.

4. If correctly identified, *Anagroidea*, *Eustochus*, and perhaps *Eoeustochus* would be the first fossil recordsof genera belonging to the extant tribe Mymarini *sensu* Annecke and Doutt (1961), likely the most derived lineage in Mymaridae.

## Supplementary Material

XML Treatment for Eoeustochus

XML Treatment for Eoeustochus kishenehn

XML Treatment for Eoeustochus borchersi

XML Treatment for Gonatocerus

XML Treatment for Gonatocerus kootenai

XML Treatment for Gonatocerus rasnitsyni

XML Treatment for Gonatocerus greenwalti

XML Treatment for Eoanaphes

XML Treatment for Eoanaphes stethynioides
